# Depolarization block in substantia nigra pars reticulata neurons drives dystonia in a mouse model

**DOI:** 10.64898/2026.09.15.751818

**Published:** 2026-09-21

**Authors:** Olivia K. Barnhill, Kasandra Romo, Alexandra B. Nelson

## Abstract

Dystonia is a common movement disorder characterized by involuntary co-contraction of antagonist muscles, resulting in abnormal postures. The brain often appears grossly normal, suggesting dysfunctional neural activity as a cause. The basal ganglia are implicated in dystonia, but the causal patterns of neural activity remain unknown. We used a transgenic mouse model of human paroxysmal nonkinesigenic dyskinesia (PNKD), in which ethanol triggers dystonic attacks, to test the role of the substantia nigra pars reticulata (SNr), the primary output nucleus of the basal ganglia in rodents. The firing of SNr neurons was profoundly reduced *in vivo* during dystonic attacks. Multiple physiological approaches indicated that in PNKD mice, ethanol leads to depolarization block in SNr neurons. Indeed, pulsatile optical activation of inhibitory striatal inputs to SNr rescued firing and attenuated dystonia. In line with theories of basal ganglia dysfunction, these results suggest reduced activity in basal ganglia output nuclei causes dystonia.

## Full Text Availability

The license terms selected by the author(s) for this preprint version do not permit archiving in PMC. The full text is available from the preprint server.

